# Molecular Epidemiology of Nontuberculous Mycobacteria Isolates from Clinical and Environmental Sources of a Metropolitan City

**DOI:** 10.1371/journal.pone.0114428

**Published:** 2014-12-08

**Authors:** Ali Akbar Velayati, Parissa Farnia, Mohadese Mozafari, Donya Malekshahian, Shima Seif, Snaz Rahideh, Mehdi Mirsaeidi

**Affiliations:** 1 Mycobacteriology Research Centre, National Research Institute of Tuberculosis and Lung Disease (NRITLD), Shahid Beheshti University of Medical Sciences, Tehran, Iran; 2 Division of Pulmonary, Critical Care, Sleep and Allergy, University of Illinois at Chicago, Chicago, Illinois, United States of America; Cambridge University, United Kingdom

## Abstract

**Introduction:**

While NTM infection is mainly acquired from environmental exposure, monitoring of environmental niches for NTM is not a routine practice. This study aimed to find the prevalence of environmental NTM in soil and water in four highly populated suburbs of Tehran, Iran**.**

**Material and Methods:**

A total of 4014 samples from soil and water resources were collected and studied. Sediments of each treated sample were cultured in Lowenstein-Jensen medium and observed twice per week for growth rate, colony morphology, and pigmentation. Colonies were studied with phenotypic tests. Molecular analysis was performed on single colonies derived from subculture of original isolates. Environmental samples were compared with 34 NTM isolates from patients who were residents of the study locations.

**Results:**

Out of 4014 samples, mycobacteria were isolated from 862 (21.4%) specimens; 536 (62.1%) belonged to slow growing mycobacteria (SGM) and 326 (37.8%) were rapid growing mycobacteria (RGM). The five most frequent NTM were *M. farcinogens* (105/862; 12.1%), *M. fortuitum* (72/862; 8.3%), *M. senegalense* (58/862; 6.7%), *M. kansasii* (54/862; 6.2%), and *M. simiae* (46/862; 5.3%*)*. In total, 62.5% (539/862) of mycobacterial positive samples were isolated from water and only 37.4% (323/862) of them were isolated from soil samples (P<0.05). Out of 5314 positive clinical samples for mycobacteria, 175 (3.2%) isolates were NTM. The trend of NTM isolates increased from 1.2% (13 out of 1078) in 2004 to 3.8% (39 out of 1005) in 2014 (P = 0.0001). The major clinical isolates were *M. simiae* (51; 29.1%), *M. kansasii* (26; 14.8%), *M. chelonae* (28; 16%), and *M. fortuitum* (13; 7.4%).

**Conclusions:**

Comparing the distribution pattern of environmental NTM isolates with clinical isolates suggests a possible transmission link, but this does not apply to all environmental NTM species. Our study confirms an increasing trend of NTM isolation from clinical samples that needs further investigation.

## Introduction

Nontuberculosis mycobacteria (NTM) or "mycobacteria other than tuberculosis" are free-living saprophytes that are found in water, soil, animals and dairy products [1], [2]. NTM was not accepted as human pathogens until 1950s, but human pathogenicity of these bacteria is well recognized now [3]. More than 150 NTM species have been described and new species continue to be identified [4].

The incidence and mortality rates of NTM infections have been increasing all over the world in the last decade [5], [6]. NTM infections are rarely spread from person to person contact and mainly acquired from environmental exposure [7], [8]. Therefore, the local environmental distribution of NTM species is an important key to predicting NTM species isolates from patients.

While monitoring of environmental niches for NTM is not a routine practice, the strong association between geography and acquisition of infection makes the investigation of NTM distribution crucial in each country. At present, there is little data representing the incidence of NTM infections in the metropolitan cities. Limited studies showed the occurrence of NTM in respiratory samples and scant reports discussed the prevalence of NTM in an urban environment [9]–[11]. To address this lack of information, we evaluated the prevalence of environmental NTM in soil and water in four highly populated suburbs of Tehran, Iran**.** To find out any correlation between environmental and clinical NTM, NTM isolates from patients who were residents of study locations were reviewed.

## Materials and Methods

This study was reviewed and approved by the Institutional Review Board of National Research Institute of Tuberculosis and Lung Diseases in Iran (approval number of MRC-2011/023/a). Before starting the project, the Municipality Department of all selected suburbs were informed about the project.

### Bacterial strains

In this study, 28 reference strains (gifted by the National Institute of Public Health and the Environment, Bilthoven, the Netherlands), 862 environmental NTM isolates and 175 clinical NTM isolates were evaluated.

### Environmental Sample collection and preparation

In total, 4014 soil and water samples were collected from four suburbs of Tehran including Robat Karim (27 km southwest of Tehran downtown), Firuzkuh (147 km northeast of Tehran downtown), Shahr-e-Ray (14 km southeast of Tehran downtown) and Varamin (35 km southeast of Tehran downtown). At least 1 sample per 100 square meters in each city was taken. In Shahr-e-ray and Varamin the number of collected samples was more because the studied area was larger. Approximately 6 grams of soil were collected from depth of 3–5 cm and suspended in 50 ml sterile tube and processed as previously described method by Portaels *et al.* [12] Briefly, 0.5 gram of soil (wet weight) was suspended in 0.2% malachite green (5 ml) and cycloheximide (1 ml at 500 µg ml^−1^). After vigorous shaking, one mole of NaOH was added to its supernatant and kept at room temp for 30 minutes. The mixture was centrifuged at 2000 g for 15 min, and oxalic acid (10 ml of 5%W/V) added to its sediments. The centrifugation was repeated for 15 minutes and the sediments inoculated onto Lowenstein-Jensen (L.J) culture media. For water samples, 50–100 ml of water from diverse water sources containing 290 samples from damp water, 260 from tap water, and 1396 from running water on raceway system were collected (table 1). In this study the damp water is referred to any stack water which has been formed because of raining, or house hold–passage, where as the raceway system is a series of cement cannels [with 10–70 m length and 20–70 cm width] that are used for transporting the waste water or sustainable rain water to central recycling and treatment sector. The collected water samples were first decontaminated with etylpyridinium chloride (CPC; final concentration of 0.05%) for 30 minutes and then digestion was performed using standard protocol [13]–[15]. Sediments of each treated sample were used to prepare a Ziehl-Neelsen smear and were cultured in Lowenstein-Jensen medium. For culture, the sediments were inoculated (200 µl of sediments/per tubes) in three LJ mediums and incubated at 37°C, 25°C and 42°C for 12 weeks. The inoculated cultures were observed twice per week for growth rate, colony morphology, and pigmentation. Colonies were studied for acid fastness and phenotypic tests (niacin, nitrate, catalyse, iron uptake and arysulfatase activity). Molecular analysis was performed on single colonies derived from subculture of original isolates as previously described [16].

**Table 1:** Shows the sources of water sample collected in different location.

Name of locations	Water samples (number = 1946)			Total
	Damp water	Tap water	Running water from race way	
Robat-Karim	38(16%)	30(12%)	181(73%)	249
Firouz-kooh	42(10%)	53(12%)	365(80%)	460
Shahre-Rey	107(19%)	80(14%)	387(68%)	574
Varamin	103 (16%)	97(15%)	463(70%)	663
Total	290	260	1396	1946

### DNA extraction

Chromosomal DNA was extracted by phenol-chloroform method [16]. The total DNA was precipitated using isopropanol and was re-dissolved in an appropriate volume of double-distilled water.

### Genotyping of the isolates

The isolates were characterized using both 16S-23S RNA and hsp65 genes spacer PCR-RFLP. For 16S-23S RNA, a set of primers (SP1 & SP2) was used to amplify a 200–350 bp fragment (SP1: 5′ ACC TCC TTT CTA AGG AGC ACC-3 and SP2: 5′ GAT GCT CGC AAC CAC TAT CCA-3′). The amplification was accomplished by an initial denaturation at 94°C for 5 min, and 30 cycles at 94°C for 30 second, at 56°C for 1 min, at 72°C for 40 second, followed by an extension at 72°C for 10 min [17]. The amplified products were digested with Hae*III* and C*foI* restriction enzymes and electrophoresed on 8% polyacrylamide gel. For hsp65 gene, a PCR reaction was amplified in 50 µl mixtures containing 4 pmol of specific primers (TB15; 5′-CGT AYG ACG AAG AGG CCC GT- 3′ and TB17; 5′-WAS GGR TCC TCS AGG ACS GC-3′), 1 µl deoxynucleotide triphosphates, 1.5 µl Mgcl, 0.25 µl (1U) Taq polymerase, 2.5 µl (1%) DMSO, 5 µl PCR Buffer, and 5 µl (20 ng) of extracted DNA. The reaction mixture was subjected to 30 amplification cycles (20 sec at 95°C, 1 min at 60°C, 40 sec at 72°C) followed by a 5 min extension at 95°C. The PCR product of the first step (470 bp) was used for the second amplification using primers TB11 (5′-ACC AAC GAT GGT GTG TCC AT-3′) and TB12 (5′-CTT GTC GAA CCG CAT ACC CT), which produced a segment of 439 bp [16]. PCR products were digested by 5 U of restriction enzyme HaeIII and BstEII for 24 hours at 37°C. The pattern of digested products was analyzed using 8% polyacrylamide gel. Species identification was performed using algorithm proposed by Roth *et al* and Telenti *et al*. [18], [19]

### Patients population

Between 2004 and 2014, 42,000 clinical samples from suspected TB patients were referred to National Reference TB laboratory (NRL). Mycobacterial species were isolated from 5314 (13%) samples. Out of 5314, 175 (3.2%) were reported NTM. 34 (11%) patients were residents of the study locations; 7 were from Robat Karim, 5 from Firuzkuh, 9 from Shahr-e-Ray and 13 from Varamin. 39 (22%) NTM isolates belonged to patients residing in the other suburbs of Tehran and 102 (58%) patients were residents of other provinces of Iran. If the patient had multiple longitudinal sampling, only the first set of samples was included into the study.

### DNA extraction and Species identification from AFB positive cultures

The DNA was extracted from heat inactivated colonies suspension using Qiagen DNA Extraction kit (QIAGEN, Hilden, Germany). Species identification was performed by using both hsp65 and 16S-23S RNA gene spacer PCR-RFLP [18]–[20].

## Results

### NTM isolation from soil and water

**Figure 1:**
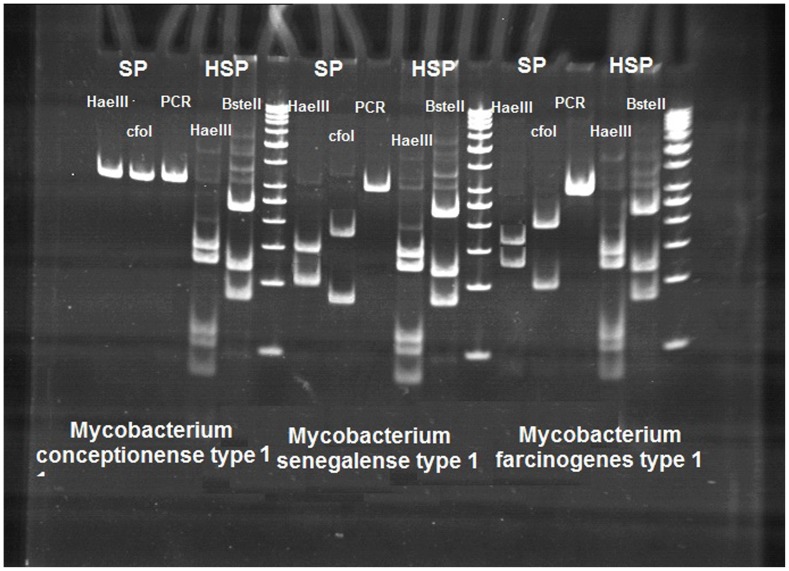
Comparison of HaeIII and BstEII RFLP patterns from isolates of *M. conceptionense*, *M. senegalense* and *M. farcinogenes* isolates from soil and water. As it shown, digested pattern of PCR product by SP primer was different in these species. No digested pattern formed in *M. conceptionense* after digestion with HaeIII and cfoI; whereas the digested pattern for *M. farcinogenes* was 190–110 bp and 168 –132 bp, respectively. The pattern for *M. senegalense* was 148–109 bp, and 174–72 bp. But the digested pattern of PCR product by HSP primer produce single pattern i.e., 234-120-80 bp with BsteIII and 140-125-60-55 bp with HaeIII restriction enzyme.

In total, 480 water and soil samples from Robat Karim, 994 from Firukuh, 1172 from Shahr-e-ray and 1368 from Varamin were collected and analyzed. Acid-fast bacteria were isolated from 862 (21.4%) samples, 1861 were AFB smear and culture negative (46.3%) and the remaining 1291 (32.1%) samples were contaminated (Table 2). Contaminated cultures were mostly smear negative (1002 out of 1291 samples; 78%) or were positive but we could not isolate the pure culture (289 out of 1291 samples; 23%). From 862 mycobacterial positive samples, 536 (62.1%) were slowly growing mycobacteria (SGM) and 326 (37.8%) strains were rapid growing mycobacteria (RGM). The most frequently isolated species amongst the SGM were *M. farcinogens* 105 (28.8%), *M. kansasii* 54 (12.7%), *M. simiae* 46 (10.8%) and *M. gordonae* 42 (9.9%). Among RGM, *M. fortuitum* 72 (22.0%), *M. senegalense* 58 (17.7%), *M. parafortuitum* 44 (13.4%), *M. chelonae* 24 (7.3%), and *M. conceptionense* 20 (6.1%) had higher frequencies. Comparison of HaeIII and BstEII RFLP patterns from isolates of *M. conceptionense*, *M. senegalense* and *M. farcinogenes* isolates from soil and water is shown in figure 1.

**Table 2:** Samples from water and soils in different study locations.

Name of locations	Water samples (Numbers = 1946)			Soil samples (Numbers = 2068)			Total
	Positive	Negative	Contaminated	Positive	Negative	Contaminated	
Robat-Karim	105(21.8%)	104(21.9%)	40(8.3%)	64(13.3%)	93(19.3%)	74(15.4%)	480
Firouz-Kooh	138(13.8%)	208(20.9%)	114(11.4%)	70(7.0%)	134(13.4%)	330(33.1%)	994
Shahre-Rey	154(13.1%)	314(26.7%)	106(9.0%)	104(8.8%)	240(20.0%)	254(21.6%)	1172
Varamin	142(10.5%)	472(39.9%)	49(3.6%)	85(6.2%)	296(21.9%)	324(24.0%)	1368
Total	539(27.6%)	1098(56.4%)	309(15.8%)	323(15.6%)	763(36.8%)	982(47.4%)	4014

Thirty-two isolates (22 from SGM and 10 from RGM) were either not identifiable (15, 1.7%) or had unknown patterns (17, 1.9%). Additionally, 95 (17.7%) isolates in SGM belonged to M.TB complex. These strains (M.TB complex & unidentifiable; 127) were excluded from the study and investigated separately (Table 3). Overall, 62.5% (539/862) of mycobacterial positive samples were isolated from water and 37.4% (323/862) were isolated from soil (P<0.05). In addition, there was significant difference between the number of positive water samples in Robat-Karim vs. other geographical locations (P<0.00001). The same difference was observed in the number of positive soil samples between Robat-Karim vs. other locations (P<0.00001).

**Table 3:** The frequency of slow growing mycobacteria and rapid growing mycobacteria species in environmental samples.

**Rapid Growing Mycobacteria = 326**		**Slow Growing Mycobacteria = 536**	
**Identified RGM = 316 (96.9%)**		**TB complex = 95 (18%)**	
**Unidentified RGM = 10 (3.0%)**		**Identified SGM = 419 (79%)**	
		**Unidentified SGM = 22 (5%)**	
*M.fortuitum (type 1, 2,4,5,6,8)*	72 (22.7%)	*M.farcinogenes type 1*	105 (28.8%)
*M.senegalense (type 1& 2)*	58 (18.3%)	*M.kansasii (type 4 &5)*	54 (12.7%)
*M.parafortuitum(type 1&2)*	44(13.9%)	*M.simiae (type1, 3 &5)*	46 (10.7%)
*M.chelonae (type 1)*	24 (7.5%)	*M.gordonae (type 1,4 &8)*	42 (9.9%)
*M.conceptionense type 1*	20 (6.3%)	*M.scrofulaceum type 1*	30 (7.0%)
*M.abscessus type1*	12 (3.7%)	*M.marinum type1*	20 (4.7%)
*M.aurum type 1*	10 (3.1%)	*M.avium type1*	20 (4.7%)
*M.mucogenicum type 1*	10 (3.1%)	*M.terrae (type 1,3 &4)*	12 (2.8%)
*M.brumae type 1*	10 (3.1%)	*M.malmoense type 1*	12 (2.8%)
*M.phlei type1*	8 (2.5%)	*M.parmense type1*	6 (1.4%)
*M.obuense type1*	6 (1.8%)	*M.sherrisii type 1*	6 (1.4%)
*M.poriferae type 1*	6 (1.8%)	*M.bohemicum type 1*	6 (1.4%)
*M.neoaurum type1*	6 (1.8%)	*M.doricum type1*	6 (1.4%)
*M.novocastrense type 1*	4(1.2%)	*M.intermedicum type 1*	6 (1.4%)
*M.rhodesia type1*	4(1.2%)	*M.szulgai type 1*	6 (1.4%)
*M.peregrinum(type 1,3)*	4(1.2%)	*M.bohemicum type 1*	6 (1.4%)
*M.aurum type 2*	4(1.2%)	*M.botniense type1*	4 (0.94%)
*M.confluentis type 1*	4(1.2%)	*M.nonchromogenicum type 1*	4 (0.94%)
*M.fluorantherivorans type1*	4(1.2%)	*M.palustre type 1*	4 (0.94%)
*M.smegmatise type 1*	4(1.2%)	*M.parascrofulaceum type 1*	4 (0.94%)
*M.gadium type 1*	2(0.6%)	*M.nonchromogenicum type 1*	4 (0.94%)
		*M.triviale type 1*	4 (0.94%)
		*M.cookii type 1*	4 (0.94%)
		*M. genavense type 1*	4 (0.94%)
		*M.flavescens type 1*	4 (0.94%)

All of *M. intermedicum* (6/6; 100%), *M. gadium* (2/2; 100%), and majority of *M. simiae* (37/46; 80.4%) were isolated from soil. In contrast, all *M. botniense* (4/4; 100%), *M.cookii* (4/4; 100%), and *M. abscessus* (12/12; 100%), and the majority of *M. senegalense* (54/58; 93%), *M. Marinum* (18/20; 90%), *M. brumae* (10/10; 90%), *M. szulgai* (5/6; 83%), *M. avium* (16/20; 80%) and *M. parafortuitum* (25/44; 56%) were isolated from water.

### NTM isolates from patients

Out of 5314 patients' samples that mycobacteria were isolated in the last 10 years, NTM was isolated from 175 (3.2%) patients. The trend of NTM isolates increased from 1.2% (13 out of 1078) in 2004 to 3.8% (39 out of 1005) in 2014 (P = 0.0001). Among clinical isolates, 119 (68%) isolates were SGM and 56 (32%) were RGM. (Tables 4) The most frequent SGM were *M. simiae* 51 (42.8%), followed by *M. kansasii* 26 (21%) and *M.intracellular* 10 (8.4%). The most common isolated species in rapid growing mycobacteria group were *M. chelonae* 28 (50%), *M. fortuitum* 13 (23.2%), *M. abscessus type 1* 9 (16.0%) and *M. parafortuitum* 10 (17.8%).

**Table 4:** The frequency of NTM species in clinical isolates from 2004–2014.

Rapidly Growing NTM		Slowly Growing NTM	
N = 56		N = 119	
*M. chelonae (type 1)*	28 (50.0%)	*M. simiae (type1)*	51(42.8%)
*M. fortuitum (type 1, 2,4,5,6,8)*	13(23.2%)	*M. kansasii (type 4 &5)*	26(21.8%)
*M. abscessus type1*	9(16.0%)	*M. intracellular type 1*	10 (8.4%)
*M. parafortuitum(type 1&2)*	6(10.7%)	*M. gordonae (type 1,4 &8)*	7 (5.8%)
		*M. avium*	7(5.8%)
		*M. genavense type 1*	3 (2.5%)
		*M. scrofulaceum type 1*	3 (2.5%)
		*M. marinum type1*	3 (2.5%)
		*M. terrae (type 1,3 &4)*	2 (1.6%)
		*M. flavescens type 1*	2 (1.6%)
		*M. szulgai type 1*	2 (1.6%)
		*M. triviale type 1*	1 (0.84%)
		*M. sherrisii type 1*	1(0.84%)
		*M. branderi*	1(0.84%)
		*M. farcinogenes*	1(0.84%)

### NTM species comparison between isolates from the environment and patients

In comparison between the environmental and clinical isolated species *M. senegalense, M. conceptionense* species were not isolated from clinical samples. Out of 34 subjects who were living in the study locations; SGM were isolated from 24 (70.5%) including *M. simiae* 18 (75%); *M. kansasii* 4 (16.6%); and *M. gordonae 2* (8.3%). RGM were isolated from 10 (29.4%) patients including *M. chelonae* 6 (60%) and *M. fortuitum* 4 (40%) (Table 5).

**Table 5:** The frequency of NTM species from clinical samples in different Tehran's suburb.

Name of locations	Most frequent RGM. N (%)	Most frequent SGM. N (%)
Robat-Karim N = 10	*M. fortuitum* 2(20) *M. chelonae* 2(20)	
Firouz-kooh N = 10	*M. fortuitum* 1(10) *M. chelonae* 1(10)	*M. simiae* 6(25.0) *M. gordonae* 2(8.3)
Shahre-Rey N = 8	*M. chelonae* 3(30)	*M. simiae* 4(16.6) *M. kansasii* 1(4.1)
Varamin N = 6	*M. fortuitum* 1(10)	*M. simiae* 3(12.5) *M. kansasii* 2(8.3)

**Figure 2:**
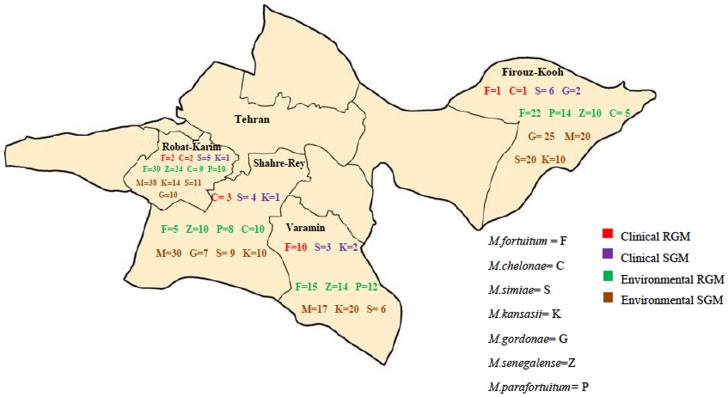
Geographic distribution of the most common NTM species isolated from clinical and environmental samples.

The prevalence of NTM strains isolates from each location were similar except for *M.kansasii* that was higher in Robat-karim and Varamin (10.5%) than two other locations; Firukuh 1.9% (4/208) and Share-ray 3.1% (8/258) (p<0.05) (Table 6). In total, the frequency of SGM was more than RGM in all the studied places (P<0.05). Figure 2 shows the geographic distribution of the most common NTM species isolated from clinical and environmental samples.

**Table 6:** The frequency of NTM species form environmental samples in different Tehran's suburb.

Name of locations	Most frequent RGM		Most frequent SGM	
**Robat-Karim N = 169**	*M. fortuitum*	25(14.9%)	*M.scrofulaceum*	7(4.1%)
	*M. senegalense*	19(11.2%)	*M.marinum*	6(3.5%)
	*M. parafortuitum*	10(5.9%)	*M.avium*	5(2.9%)
	*M. conceptionense*	5(2.9%)	*M.farcinogenes*	27(15.9%)
	*M. abscessus*	3(1.7%)	*M.kansasii*	18(10.6%)
	*M. chelonae*	7(4.1%)	*M.simiae*	10(5.9%)
			*M.gordonae*	8(4.7%)
**Firouz-kooh N = 208**	*M.fortuitum*	9(4.3%)	*M.scrofulaceum*	1(3.8%)
	*M.senegalense*	12(5.7%)	*M.marinum*	8(3.8%)
	*M.parafortuitum*	14(6.7%)	*M.avium*	6(2.8%)
	*M.conceptionense*	4(1.9%)	*M.farcinogenes*	27(12.9%)
	*M.abscessus*	4(1.9%)	*M.kansasii*	4(1.9%)
	*M.chelonae*	5(2.4%)	*M.simiae*	18(8.6%)
			*M.gordonae*	17(8.1%)
**Shahre-Rey N = 258**	*M.fortuitum*	18(6.9%)	*M.scrofulaceum*	15(5.8%)
	*M.senegalense*	22(8.5%)	*M.marinum*	4(1.5%)
	*M.parafortuitum*	7(2.7%)	*M.avium*	4(1.5%)
	*M.conceptionense*	10(3.8%)	*M.farcinogenes*	26(10.0%)
	*M.abscessus*	3(1.1%)	*M.kansasii*	8(3.1%)
	*M.chelonae*	5(1.9%)	*M.simiae*	11(4.2%)
			*M.gordonae*	9(3.4%)
**Varamin N = 227**	*M.fortuitum*	20(8.8%)	*M.scrofulaceum*	6(2.6%)
	*M.senegalense*	5(2.2%)	*M.marinum*	2(0.8%)
	*M.parafortuitum*	13(5.7%)	*M.avium*	5(2.2%)
	*M.conceptionense*	5(2.2%)	*M.farcinogenes*	25(11.0%)
	*M.abscessus*	2(0.8%)	*M.kansasii*	24(10.5%)
	*M.chelonae*	3(1.3%)	*M.simiae*	7(3.0%)
			*M.gordonae*	8(3.5%)

RGM: rapid growing mycobacteria, SGM: slow growing mycobacteria.

## Discussion

We demonstrated that *M. farcinogenes* and *M. fortuitum* are the most common inhabitant species in water and soil and *M. simiae* and *M. chelonae* are the most common isolated species from patients living in the same locations. Also, it is noticeable that 10-27% of water samples and 6–15% of soil samples of these Tehran suburbs are inhabited with NTM. Slow growing mycobacteria are found as the most common isolatable species from environmental samples.

Interestingly, *M. farcinogenes* was the most common mycobacteria isolated from environment in the current study. *M. farcinogenes* is the causal agent of bovine farcy, a chronic and progressive disease of the skin and lymphatics of zebu cattle. Zebu cattle are cows with long horns that are the most farmed cows in Iran [21]. There is no another report on isolating or *M. farcinogenes* from environmental samples [22], but it has been isolated rarely from clinical specimens [23]. This is the first report that highlights *M. farcinogenes* isolation from environmental samples from Iran. In contrary to environmental samples, *M.farcinogenes* was rarely isolated from clinical isolates in our study. Out of 175 patients with NTM isolates, *M.farcinogenes* was identified only from one patient. Given the high prevalence of *M. farcinogenes* in Tehran suburbs and its potential pathogenicity to cattle and humans, further epidemiological investigation is needed.

In the present study, *M. simiae* was the most common clinical isolate among the slow growing mycobacteria. *M. simiae* can cause infection is different organs, particularly lungs. While *Mycobacterium avium complex* is the most common isolated NTM from clinical samples in the US, the isolation of *M. simiae* is more common in Middle Eastern patients [24]–[26]. It has been proposed that people from Middle East region have higher susceptibility to this mycobacterium [25]. There are a few reports on virulence of *M. simiae* for humans in Iran [9], [27], [28]. Baghaei and coworkers recently described the clinical and radiological characteristics of pulmonary *M. simiae* infection among Iranian patients [29].

*M. chelonae* and *M. fortuitum* were the most common RGM isolated from the clinical samples. Hashemi *et al.* found that *M. fortuitum* was the most common cause of NTM diseases in Isfahan (a city located in the center of Iran) [30]. They evaluated 92 subjects with NTM diseases and concluded that rapid growing mycobacteria were the most common cause of infection in immunocompromised patients.

This study has several limitations. The primary limitation of this study is relatively small clinical sample size. There were 34 cases from residents of the study locations. Genetic sequencing to assess genetic similarity between the environmental and clinical samples was not performed. Despite these limitations, this is the first study that has evaluated the environmental NTM in a large scale.

In conclusion, there is a strong evidence to suggest a correlation between the pattern of environmental NTM distribution and clinical infection, but this does not apply to all environmental NTM species. Some other factors rather than environmental distribution can have influence in acquiring NTM infection that should be further investigated. Our study confirms an increasing trend of NTM isolation from clinical samples that needs further investigation.
